# Preparation, Shelf, and Eating Quality of Ready-to-Eat “Guichang” Kiwifruit: Regulation by Ethylene and 1-MCP

**DOI:** 10.3389/fchem.2022.934032

**Published:** 2022-07-13

**Authors:** Han Yan, Rui Wang, Ning Ji, Sen Cao, Chao Ma, Jiangkuo Li, Guoli Wang, Yaxin Huang, Jiqing Lei, Liangjie Ba

**Affiliations:** ^1^ College of Food and Pharmaceutical Engineering, Guiyang University, Guiyang, China; ^2^ Tianjin Key Laboratory of Postharvest Physiology and Storage of Agricultural Products, National Engineering and Technology Research Center for Preservation of Agricultural Produce, Tianjin, China; ^3^ Fruit Industry Development Service Centre for Xiuwen County, Guiyang, China

**Keywords:** instant kiwifruit, edible window, ethylene, 1-methylcyclopropene, regulation

## Abstract

The acceptance of kiwifruit by consumers is significantly affected by its slow ripening and susceptibility to deterioration. Ready-to-eat “Guichang” kiwifruit and its preparation technology were studied by the regulation of ethylene and 1-MCP. Harvested kiwifruits were treated with 100–2000 μl L^−1^ethylene for 36 h (20°C) and then treatment with 0–0.5 μl L^−1^ 1-MCP. The results showed that the preservation effect of 0.5 μl L^−1^ 1-MCP is inefficient when the soluble solid content of kiwifruit exceeded 15%. The ethylene-treated fruits reached an “edible window” after 24 h, but a higher concentration of ethylene would not further improve ripening efficiency, while the optimal ethylene concentration was 250 μl L^−1^. Moreover, after 250 μl L^−1^ ethylene treatment, 0.5 μl L^−1^ 1-MCP would effectively prolong the “edible window” of fruits by approximately 19 days. The volatile component variety and ester content of 0.5 μl L^−1^ 1-MCP-treated fruits were not different from those of the CK group. Principal component analysis and hierarchical cluster analysis indicated that the eating quality of fruits treated with 0.5 μl L^−1^ 1-MCP was similar to that of fruits treated with ethylene. Consequently, ready-to-eat “Guichang” kiwifruit preparation includes ripening with 250 μl L^−1^ (20°C, 36 h) ethylene without exceeding the 1-MCP threshold and then treated with 0.5 μl L^−1^ 1-MCP (20°C, 24 h). This study highlights the first development of a facile and low-cost preparation technology for ready-to-eat “Guichang” kiwifruit, which could reduce the time for harvested kiwifruit to reach the “edible window” and prolong the “edible window” of edible kiwifruit.

## 1 Introduction

Kiwifruit production has experienced sustained growth globally, from 3.63 million tons in 2014 to 4.34 million tons in 2019. In addition, kiwifruit occupies an important position in the fruit market (Wang S. et al., 2021). Meanwhile, China is the largest kiwifruit producer in the world, with an annual output of 2.5 million tons and a planting area of 243,000 ha (Wang et al., 2021b). The Guizhou Province is located in southwest China and is one of the main production areas of kiwifruit, but also one of the world’s three major karst landscape areas, the center of east Asia. This area is suitable for the growth of kiwifruit because of its unique geographical and climatic environment. “Guichang” kiwifruit is an independent breed, and its planting area reached over 40,000 hm^2^ (Wang et al., 2021c) in Guizhou Province. This variety is similar to “Hayward,” with green flesh, sweet taste, and high vitamin C content, which is favored by consumers and commercialized.

Kiwifruit is a climacteric fruit, which indicates the characteristics of after-ripening. Hence, it is generally harvested at a physiologically mature stage—unripe and inedible. Consumers have to wait for 6–8 days to get the kiwifruit edible. Simultaneously, kiwifruit is sensitive to ethylene, and it produces a large amount of endogenous ethylene during the ripening stage; then, it rapidly softens and decays gradually as it reaches the “edible window” stage (Burdon et al., 2017; Tilahun et al., 2020; Wang H. et al., 2021; Huang et al., 2021). Therefore, this seriously affects the storage and consumption of kiwifruit. The aforementioned two factors affect the commodity value of kiwifruit along with reducing consumers’ experience and the likelihood of repurchase. Accordingly, it is urgent and essential to develop a facile and low-cost preparation technology for ready-to-eat kiwifruit with a shorter softening time and a longer edible window time.

Ethylene is a natural gaseous plant hormone that plays a vital role in accelerating the softening and ripening of kiwifruit, thus reducing the waiting time to eat kiwifruit and promoting better flavor. Park et al. (2006) treated “Hayward” kiwifruit with 100 μg L^−1^ ethylene for 24 h at 20°C, and the firmness of the ethylene-treated fruit reached a minimum after 2 days, which was 8 days earlier than that of the untreated fruits. Lim et al. (2017) treated “Jecy” green kiwifruit with 200 μl L^−1^ ethylene for 12 h at 20°C and found that the firmness of the ethylene-treated fruit was reduced to 10 N at 2 days, which was 3 days earlier than CK. The fruit needs at least 2 days to reach the “edible window” throughout ethylene ripening. Moreover, there has been no report on the ripening efficiency of kiwifruit treated with a higher concentration of ethylene.

1-MCP is an ethylene receptor blocker, which can inhibit endogenous ethylene production and the physiological and biochemical reactions of fruit by irreversibly binding with the ethylene receptor (Gong et al., 2020). In previous studies, the 1-MCP treatment showed the effect of inhibited endogenous ethylene production, reduced respiratory rate, upregulated the expression of key enzyme genes in the ascorbic acid synthesis pathway, and promoted ascorbic acid metabolism in kiwifruit, thus enabling to delay the deterioration of fruit quality (Di Francesco et al., 2018; Huan et al., 2020; Zhang et al., 2021). However, the effect of 1-MCP on edible kiwifruit has not yet been reported.

In this study, the concept of ready-to-eat kiwifruit was proposed for the first time. The aim of the present study is to develop a kiwifruit post-harvest technology and innovate a ready-to-eat kiwifruit technology that includes 1) the threshold of 1-MCP on kiwifruit; 2) the effect of higher concentrations of ethylene (100, 250, 500, 1,000, and 2,000 μl L^−1^) on the efficiency of kiwifruit ripening; 3) the preservation effect of 1-MCP on edible kiwifruit; 4) the flavor compounds of ready-to-eat kiwifruit; and 5) the eating quality of ready-to-eat kiwifruit is completely evaluated by HCA and PCA, including the Brix-acid ratio (BAR), pulp firmness, ascorbic acid (ASA), folic acid, color, and volatile components. These findings provide a technical basis for reducing the post-harvest loss of kiwifruit and improving its commercial value.

## 2 Materials and Methods

### 2.1 Materials

Kiwifruit (*Actinidia deliciosa* cv. “Guichang”) was harvested in a kiwifruit garden, in Xiuwen County (106°40′14″ E, 26°57′35″ N) in 2020, and then immediately transported to a laboratory at Guiyang University. The average SSC of fruit was 7.0% (*n* = 18). On harvest day, all the fruits were placed at room temperature, and the callus lasted for 24 h before they were divided into three parts to study the following: 1) threshold of 1-MCP treatment; 2) effect of ethylene on fruit ripening; and 3) effect of 1-MCP on the ripe fruit. In order to simulate the kiwifruit market model in practice, a batch of fruits was stored in a controlled atmosphere container (Tianjin Lvyuan Jieneng Air Conditioning Fresh-keeping Equipment Co., LTD., LYQT-400, China) and ready to be prepared for ready-to-eat kiwifruit. The storage conditions were as follows: O_2_: 2%; CO_2_: 4.5%; 90% RH (Di Francesco et al., 2018).

### 2.2 Treatment and Assessments

#### 2.2.1 Threshold of 1-MCP Treatment

To investigate the threshold of 1-MCP on kiwifruit with different maturities, 2,400 fruits were employed and divided into six groups. Logically, the kiwifruit maturity was increased with shelf time because the kiwifruit was attributable to climacteric fruit. Herein, six groups of fruit with different maturities were obtained every 3 days. Immediately, 400 fruits were treated by 1-MCP (0.5 μl L-1, 24 h), named F0, F3, F6, F9, and F12, respectively. In comparison, the group obtained at 0 day without 1-MCP treatment was called CK. After treatment, a shelf was carried out for 12 days at 20°C, and the detection was performed at 0, 3, 6, 9, and 12 days in turn.

#### 2.2.2 Effect of Ethylene on Fruit Ripening

After 45 days of controlled atmosphere storage, 1,800 fruits were divided into six groups for the ethylene ripening experiment (300 fruits in each group). Ethylene-treated (ET) groups were placed in plastic boxes (60 L, 55*40*31.5 cm) and then injected with different volumes of ethylene (100, 250, 500, 1,000, and 2,000 μl L^−1^), while the control group was not treated by ethylene. These groups were named E1, E2, E3, E4, E5, and CK, respectively. The O_2_ and CO_2_ concentrations in the box were monitored by a headspace analyzer (Check Point II, Dansensor, Denmark) every 3 h. Ripening was terminated when the O_2_ concentration in any group was lower than 5%. After treatment, a shelf experiment was carried out for 9 days at 20°C, and the detection was performed at 1, 3, 5, 7, and 9 days. Therefore, the optimum ripening conditions were obtained.

#### 2.2.3 Effect of Ethylene on Ripe Fruit

Based on the ethylene ripening experiment results, the ripe kiwifruits were divided into three groups (450 fruits in each group) for ethylene ripening according to the optimum conditions. Then, all the groups were treated with 0, 0.25, or 0.5 μl L^−1^ 1-MCP for 24 h at 20°C. After the treatment, all fruits were stored at 4°C for 14 days and then shelved for 7 days at 20°C. Detection was performed for the fruit at 0 day, 14 days (4°C), 3 days (20°C), 5 days (20°C), and 7 days (20°C).

For GC-MS, the fruit was peeled and frozen in liquid nitrogen and stored at −80°C.

### 2.3 Physicochemical Analysis

Fruit quality was assessed for the ethylene production (EP) rate, respiration intensity (RI), decay percentage, pulp firmness, SSC, titratable acid (TA), Brix-acid ratio (BAR), h°, starch, ascorbic acid (ASA), and folic acid.

In the aforementioned three experiments, 27 fruits in each group were selected for the determination of EP and RI throughout the experiment. EP and RI were calculated for a constant nine fruits per replication. Nine fruits were placed in a 3.2 L sealed plastic box at 20°C for 2 h. A gas chromatograph was loaded with 2 ml of sample gas from the plastic box (GC-14C, Shimadzu, Tokyo, Japan). The GC condition was referred to in a previous study and was modified (Patil et al., 2019). The inlet temperature was 145°C, the cylinder temperature was 40°C, the detector temperature was 180°C, and the auxiliary heater was 235°C. The heating procedure was as follows: 40°C for 7 min, then increased to 100°C at a rate of 10°C min^−1^−100°C and maintained for 3 min.

Decay percentage was measured by counting the number of fruits decayed in each group by the method of Choi et al. (2019). Pulp firmness (*n* = 18) was measured according to Burdon et al.’s (2017) report, which used a texture analyzer (TA. XT Plus, SMS, England) fitted with a 2 mm penetrometer probe. The SSC (*n* = 18) and TA content (*n* = 3) were determined according to previous studies (Sivakumaran et al., 2016; Burdon et al., 2017). Then, BAR was calculated by SSC and TA. Pulp color (*n* = 18) was measured by a grating spectrophotometer (YS3060, 3nh Science and Technology Ltd., China) according to Patil et al.’s (2019) report. Starch, ASA, and folic acid content (*n* = 3) measurements were conducted with frozen materials, which were determined by a method based on previous studies (Johansson et al., 2005; Tavarini et al., 2008).

### 2.4 Sensory Evaluation

Sensory evaluation of kiwifruit was conducted by using a 9-point hedonic scale discussed by Nirmal et al. (2020). All samples were labeled with a three-digit code. Observations regarding the appearance, color, aroma, taste, and overall acceptability of each sample were performed by ten well-trained consumers.

### 2.5 GC-MS Analysis

Extraction of GC-MS-based volatiles was performed as described in a previous study with some modifications (Du et al., 2019). A 10 μl L^−1^ 3-octanol internal standard was prepared with n-hexane as the solvent. An internal standard of 1 μl and kiwifruit pulp, as well as 2.0 g sodium chloride and a polypropylene cap, were sealed by a polytetrafluoroethylene/silicon septum for each sample (Agilent Technologies, United States). In addition, the prepared samples were heated in a water bath at 40°C for 30 min and then loaded into an Agilent 7890B GC system (Agilent Technologies, United States) by an Agilent 76978 headspace sampler.

The GC conditions were as follows: HP-5ms Agilent quartz capillary column (30 m × 0.32 mm, 0.25 μm; Agilent Technologies, America); helium carrier gas (99.999%), 2 ml min^−1^, no shunt; injector temperature of 250°C. The initial temperature was 35°C and maintained for 3 min, increased to 45°C at a rate of 3°C min^−1^, increased to 120°C at a rate of 2°C min^−1^, increased to 240°C at a rate of 6°C min^−1^, and maintained for 6 min. The MS conditions were as follows: electron impact ionization source; electron energy: 70 eV; ionization temperature: 230°C; quadrupole temperature: 150°C; interface temperature: 280°C; and quantity scanning range: 30–500 amu.

The identification of the unknown volatile compounds was performed by the NIST 17 L library. The volatile content relative to the internal standard was calculated according to the comparison between the internal standard content and the chromatographic peak area of the volatile and the internal standard. The formula is as follows:

$$\omega_{x}=\frac{A_{x}n_{is}M_{x}}{m_{n}A_{is}}\times 10^{9},$$

where ω_x_ is the concentration of the unknown volatile (µg kg^−1^), n_is_ is the amount of the internal standard substance (g mol^−1^), M_x_ is the molar mass of the internal standard substance, A_x_ is the peak area of the unknown compound, A_is_ is the peak area of the internal standard substance, and m_n_ is the sample amount (g).

### 2.6 Statistical Analysis

Data results were statistically processed by SPSS 21.0 software (SPSS Inc., United States), and the results were expressed as the mean ± sd. Duncan multiplicity comparison was used for significance analysis (*p* < 0.05), and Origin Pro 2017 software (Origin Lab Inc., United States) was used for plotting.

## 3 Result

### 3.1 Threshold of 1-MCP Treatment

**FIGURE 1:**
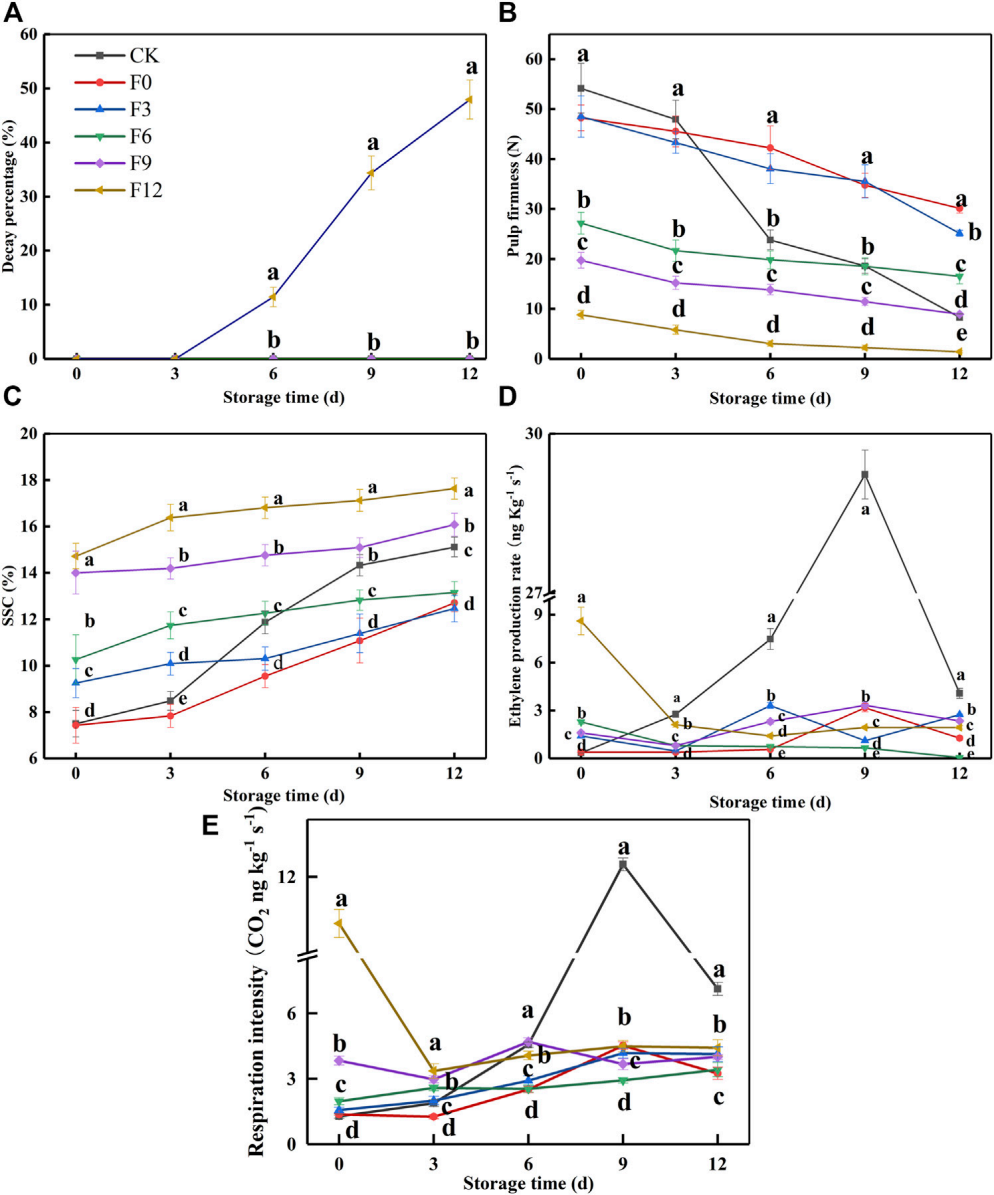
Decay percentage **(A)**, pulp firmness **(B)**, SSC **(C)**, ethylene production rate **(D)**, and respiration intensity **(E)** variations during fruit storage in the 1-MCP threshold experiment. Data are the means of three replicates ± standard deviation (*n* = 18). According to Duncan’s test, values with different letters are significantly different (*p* < 0.05).

In previous studies, kiwifruit entered the “edible window” with SSC of 14–20% and firmness of 3.1–14 N (Deng et al., 2015; Quillehauquy et al., 2020; Zhang et al., 2021). However, the fruit began to soften rapidly at this stage, so the “edible window” was shortened. Therefore, it was of great importance for ready-to-eat kiwifruit to find the preservation threshold of kiwifruit affected by 1-MCP. As shown in Figure 1A, the samples of the F0, F3, F6, and F9 groups did not decay during the shelf life. In Figures 1B and C, the fruit maturities of the CK, F0, F3, F6, F9, and F12 groups were different because they were treated separately every 3 days in turn. The CK group reached an “edible window” at 12 days (SSC: 15.11%, pulp firmness: 8.36 N). Meanwhile, the ranges of SSC and pulp firmness of F0, F3, and F6 were 12.46–13.15% and 30.12–16.55 N (Figures 1B, C), respectively. Obviously, the fruit was inedible at this stage. The F9 group was within its “edible window” from 6 days (SSC: 14.76%, pulp firmness: 13.86 N) to 12 days (SSC: 16.08%, pulp firmness: 8.98 N), with a fruit quality similar to that of the CK group at 12 days (SSC: 15.11%, pulp firmness: 8.36 N) (*p* < 0.05). According to the aforementioned SSC, firmness, and decay percentage, 1-MCP provided an obvious preservation effect on F0, F3, F6, and F9 groups. Although the SSC of the F12 group was the highest, its decay percentage increased gradually from 11.45 to 47.91% until 12 days (Figure 1A). The previous results indicated that 1-MCP had a poor preservation effect on the F12 group.

The EP and RI of the CK group gradually increased to a maximum until 9 days and then sharply decreased to 4.09 ng kg^−1^ h^−1^ and 7.11 ng kg^−1^ s^−1^ at 12 days, respectively (Figures 1C, D). In contrast, the highest EP and RI of the F0, F3, F6, and F9 groups were 2.28–3.32 ng kg^−1^ h^−1^ and 3.42–4.69 ng kg^−1^ s^−1^, respectively, which were lower than those of the CK group (29.23 ng kg^−1^ h^−1^ and 12.32 ng kg^−1^ s^−1^). The EP and RI of the F12 group decreased at first and then remained basically unchanged from 3 to 12 days. Because the climacteric of the CK group occurred at 9 days, the F12 group had undergone a climacteric before treatment with 1-MCP. In addition, the EP and RI of the F12 group at 0 day were higher than those of the CK group at 12 days, which indicated that 1-MCP was ineffective in the F12 group. Altogether, it was recommended that the threshold of 1-MCP on Guichang kiwifruit be SSC: 15%.

### 3.2 Effect of Ethylene on Fruit Ripening

**FIGURE 2:**
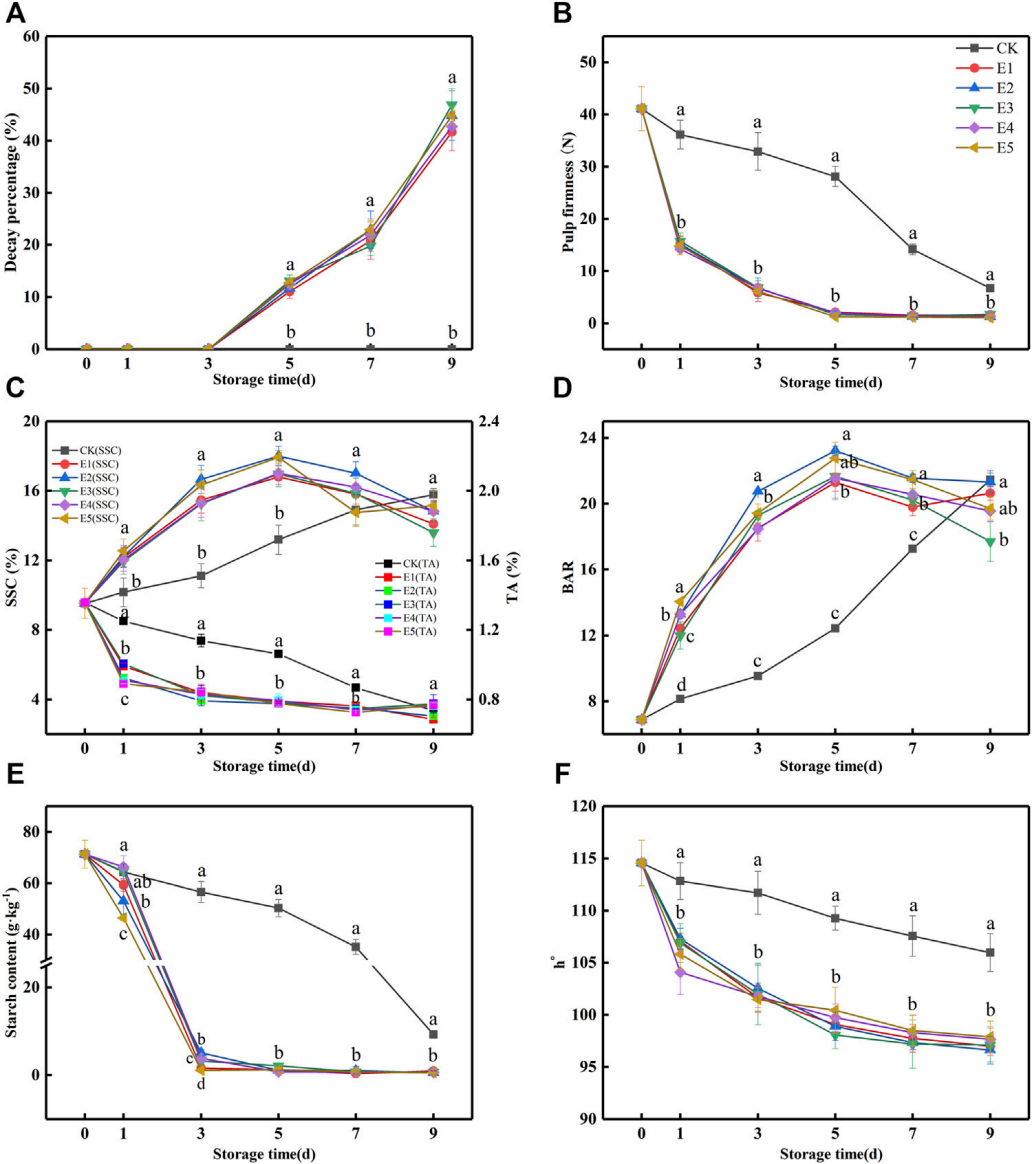
Decay percentage **(A)**, pulp firmness **(B)**, SSC and TA **(C)**, BAR **(D)**, starch **(E)**, and h° **(F)** variations during fruit storage in the ethylene ripening experiment. (CK: 0 μl L^−1^, E1: 100 μl L^−1^, E2: 250 μl L^−1^, E3: 500 μl L^−1^, E4: 1,000 μl L^−1^, and E5: 2,000 μl L^−1^). Data are the means of three replicates ± standard deviation (*n* = 18). According to Duncan’s test, values with different letters are significantly different (*p* < 0.05).

From previous studies, kiwifruit needs 2–3 days to reach the “edible window” after treatment with 100 μl kg^−1^, 100 μg L^−1^, and 200 μl L^−1^ ethylene (Park et al., 2006; Lim et al., 2017; Tilahun et al., 2020). To improve ripening efficiency, we attempt to directly ripen kiwifruit to an edible window, thus higher concentrations of ethylene (100, 250, 500, 1,000, and 2,000 μl L^−1^) and shorter ripening time were introduced. The concentrations of O_2_ and CO_2_ were monitored with a headspace analyzer every 3 h to prevent anaerobic respiration during ethylene ripening. Ripening was stopped at 36 h because the O_2_ concentration in the ET groups was lower than 5% (Supplementary Figure S1). From Figures 2B and C, after 36 h of ripening, the SSC of fruit was increased from 9.53 to 12.4%, and the pulp firmness of fruit was decreased from 41.1 to 14.2 N. It was closest to the edible window (SSC: 14–21%; pulp firmness: 3.1–14 N).

After ripening, a shelf was performed under 20°C for 9 days. First, the decay percentage of all groups is presented in Figure 2A. At 5 days, the samples from ET groups began to decay. However, decay did not occur in the CK group. According to Figure 2B, the pulp firmness of ET rapidly decreased after ripening by ethylene to a nearly edible firmness at 1 day (14.2–15.7 N), while the pulp firmness of the CK group was 36.15 N at this time point. At 3 days, the pulp firmness of the ET groups (5.84–6.83 N) reached edible firmness, while the CK group was at 9 days. Although ethylene ripening accelerated fruit softening and decay, there was no difference among the ET fruits.

From Figure 2C, ethylene accelerated the increase in SSC in the ET groups, which was significantly higher than that in the CK group at 1–5 days. The SSC of the ET groups reached the edible SSC (14–20%) at 3 days (15.26–16.66%) and increased to a maximum (16.81–18.01%) at 5 days. However, the SSC of the CK group reached a maximum (15.78%) at 9 days, which was lower than that of the ET groups. According to the SSC, decay percentage, and pulp firmness of the ET groups (Figures 2A–C), the samples reached the “edible window” at 3 days, but the decay percentage exceeded 10% at 5 days. Ethylene ripening promoted TA reduction in kiwifruit. As shown in Figure 2C, the TA of the ET groups decreased sharply at first and then slowed until 9 days. However, the reduction of CK was slower and lower than ET from 1 to 7 days. BAR, calculated by SSC and TA, was used to measure fruit taste, which was an important indicator of the taste of fruit (Figure 2D). From Figures 2C and D, the BAR of samples in all groups showed an increasing trend, followed by an increase in SSC and a decrease in TA. Clearly, the changes in SSC and TA in the ET groups were more rapid than those in the CK group. When the sample was in the “edible window” before decay, the highest BARs of the CK, E1, E2, E3, E4, and E5 groups were 21.44, 18.50, 20.74, 19.28, 18.48, and 19.42, respectively. At this stage, the highest BAR of the E2 group (3 days) was closest to that of the CK group (9 days) and higher than that of E1, E3, and E4, which indicated that the taste of fruit with 250 μl L^−1^ ethylene ripening was the most similar to that of the CK group.

Kiwifruit provides energy for its physiological and metabolic activities through the decomposition of starch, which is eventually converted into fructose and glucose to promote the rise of SSC (Lim et al., 2017). Therefore, the maturity of kiwifruit can be evaluated by the starch content. As shown in Figure 2E, the starch content of the CK and ET groups showed a downward trend during the shelf, and ET was faster than CK. At 3 days, the starch content of the ET groups was 1.11, 5.13, 3.26, 3.85, and 1.11 g kg^−1^, respectively. The E2 group was higher than E1, E3, E4, and E5, which indicated that the quality of E1, E3, E4, and E5 was poorer than that of E2. Hence, the ripening effect of E2 was the best in the ET groups.

Fruit color always directly affects the shopping experience of the consumer (Xia et al., 2021). Patil et al. stated that when kiwifruit “Hort16A” was treated with 1 μl L^−1^ ethylene at 1.5°C for 3 weeks, the h° of the treated group was significantly lower than that of CK (Patil et al., 2019). According to a prior report on kiwifruit (Deng et al., 2015), when h° was close to 90°, the sample seemed yellow, and as h° increased, the sample appeared greener. In this study, the h° of the ET groups was significantly lower than that of CK (Figure 2F). However, there were no differences between E1, E2, E3, E4, and E5. From Figure 2F, the h° of all ET groups at 1 day (104.08–107.30) was closest to that of CK at 9 days (105.98) before decay.

**FIGURE 3:**
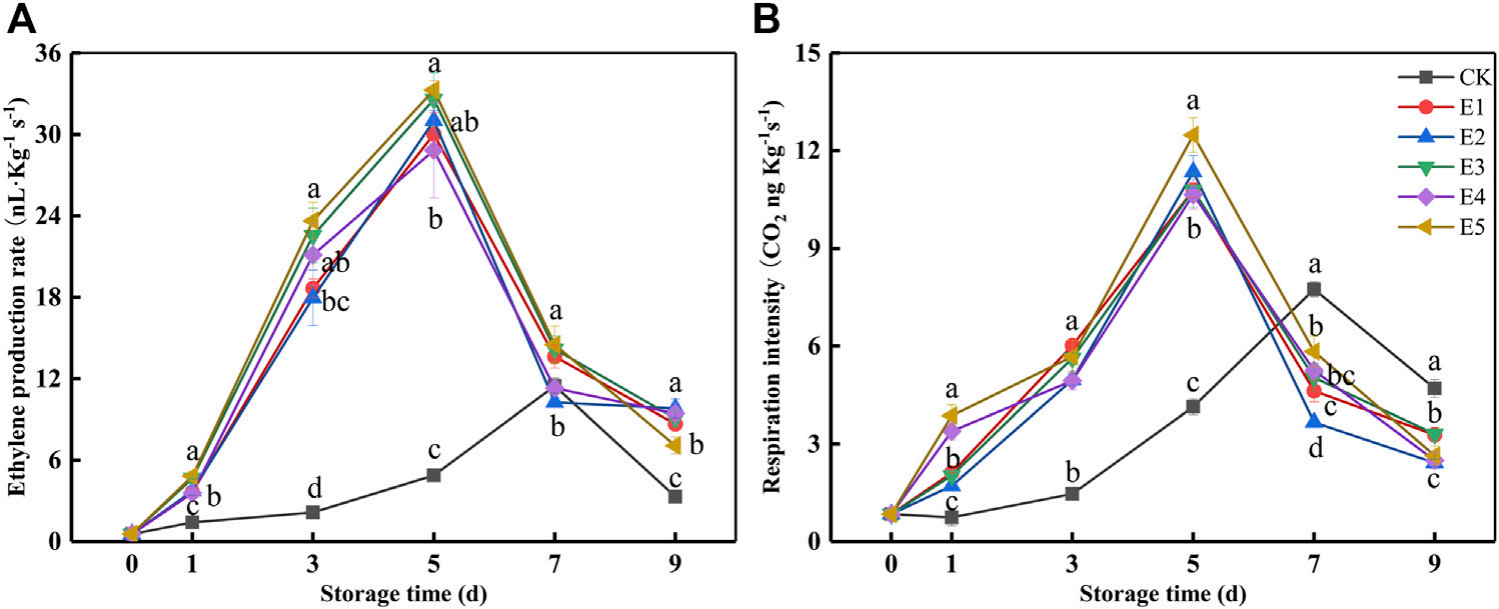
Ethylene production rate **(A)** and respiration intensity **(B)** variations during fruit storage in the ethylene ripening experiment. Data are the means of three replicates ± standard deviation (*n* = 18). Values with different letters are significantly different according to Duncan’s test (*p* < 0.05).

All the indicator results shown in Figure 2 could be interpreted by EP and RI (Figure 3) after ripening. The EP and RI of the ET groups increased, and the maximum occurred at 5 days (28.83–33.28 ng kg^−1^ s^−1^ and 10.64–12.48 ng kg^−1^ s^−1^). While the maximum of CK appeared at 7 days (11.46 μl kg^−1^ h^−1^ and 7.74 ng kg^−1^ s^−1^), it was 60.24–65.56% and 27.25–37.98% lower than ET, respectively. In Figures 2B–F and Figure 3, the pulp firmness, SSC, TA, BAR, and h° of the ET groups changed rapidly within 1–5 days, which was consistent with the changing trend of EP and RI. In addition, when the SSC, EP, and RI (Figures 2C, 3) reached the maximum, the fruit from ET groups began to decay (Figure 2A), which indicated the fruit was in senescence. Taken together, there were no differences in pulp firmness, SSC, TA, or h° among the ET groups.

### 3.3 Effect of 1-MCP on the Ripe Fruit

After the 250 μl L^−1^ ethylene ripening, the SSC of fruits reached a high level (SSC: 14%), and it reached the edible window. Subsequently, the fruits will rapidly decay without any treatment. Therefore, we proposed to prepare ready-to-eat kiwifruit with a long edible window. In the present work, the ready-to-eat kiwifruit was prepared by ethylene ripening followed by 1-MCP preservation. Herein, 1,350 ripe fruits (SSC: 14.51%) were treated with 1-MCP. The effect of 1-MCP (0 μl L^−1^; 0.25 μl L^−1^; 0.5 μl L^−1^) on the shelf life of edible kiwifruit was further investigated. In order to evaluate the shelf-life of ready-to-eat kiwifruit under the supermarket condition, a shelf-life experiment was performed at 4°C for 14 days and then at 20°C for 7 days.

**FIGURE 4:**
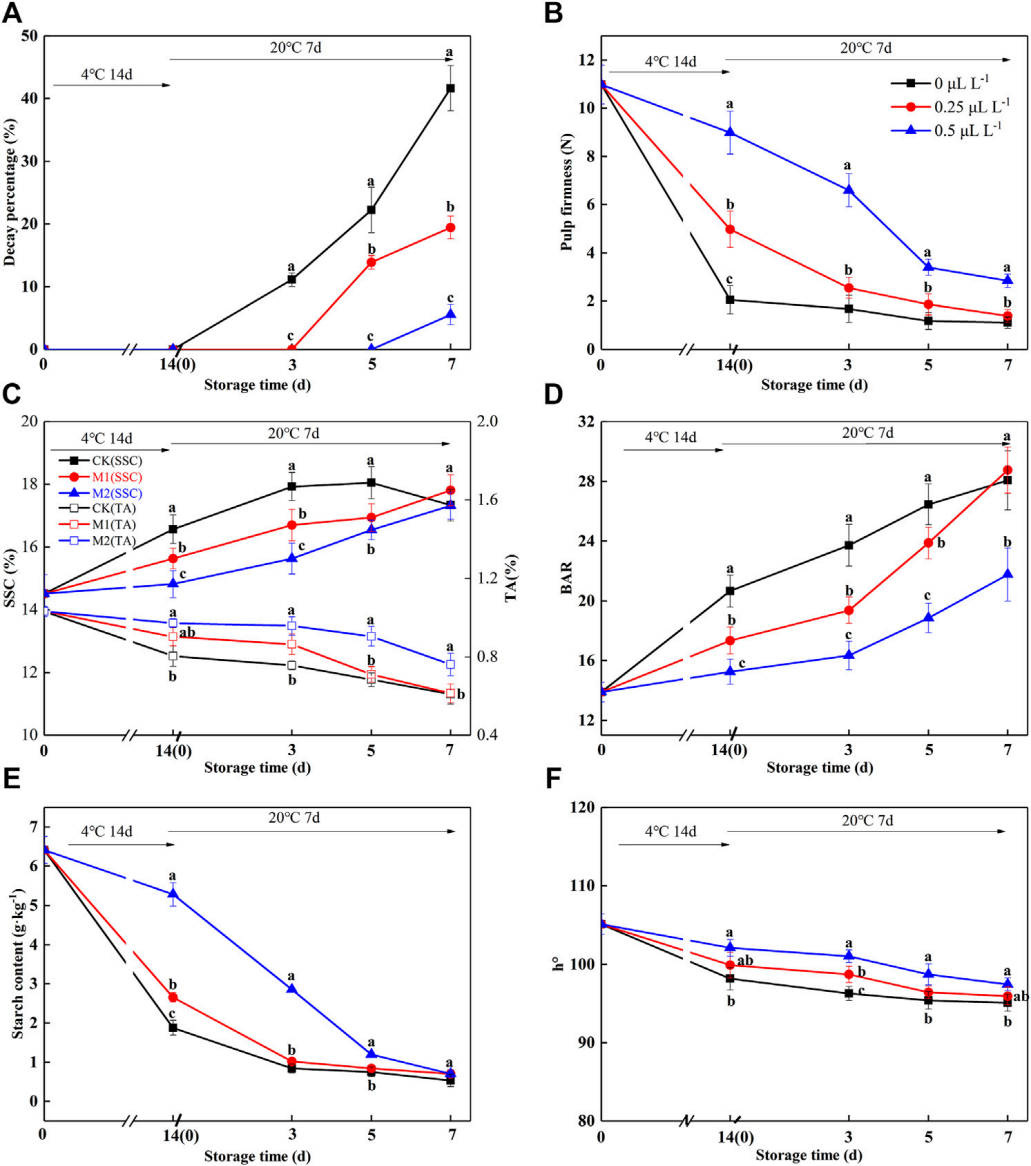
Decay percentage **(A)**, pulp firmness **(B)**, SSC and TA **(C)**, BAR **(D)**, starch **(E)**, and h° **(F)** variations during shelf life in the 1-MCP preservation experiment. Data are the means of three replicates ± standard deviation (*n* = 18). According to Duncan’s test, values with different letters are significantly different (*p* < 0.05).

From Figure 4, no decay occurred in any group during the 4°C shelves for 14 days. When samples were placed on 20°C shelves, the 0 μl L^−1^ 1-MCP group began to decay at 3 days, and the decay percentage was 11.87%. The 0.25 and 0.5 μl L^−1^ 1-MCP groups began to decay at 5 days or 7 days, and the decay percentages were 11.88 and 14.58%, respectively.

To reduce the risk of a negative consumer experience, prior to decay, all groups underwent sensory evaluation, nutrition, and volatile component analysis. The sensory evaluation results (appearance, color, aroma, taste, overall) were presented in Table 1. With time, although the values of “color” and “appearance” decreased, “aroma” and “taste” increased, and there was no difference in the “overall” value before decay.

**TABLE 1:** Sensory score of kiwifruits.

Shelf time	Treatment	Sensory Attribute				
		Appearance	Color	Aroma	Taste	Overall
0 d	0 μl L^−1^	8.4 ± 0.6	7.5 ± 0.5	5.6 ± 0.6	5.2 ± 0.4	5.7 ± 0.5
4°C	0 μl L^−1^	7.4 ± 0.6^a^	7.1 ± 0.5^a^	6.6 ± 0.6^a^	6.8 ± 0.4^a^	7.7 ± 0.4^a^
14 days	0.25 μl L^−1^	8.1 ± 0.4^a^	7.2 ± 0.5^a^	5.7 ± 0.5^a^	5.9 ± 0.5^a^	6.3 ± 0.6^b^
	0.5 μL/L	8.2 ± 0.4^a^	7.2 ± 0.6^a^	5.7 ± 0.4^a^	5.5 ± 0.6^b^	6.2 ± 0.4^b^
20°C 3 days	0 μl L^−1^	—	—	—	—	—
	0.25 μl L^−1^	7.2 ± 0.5^a^	7.0 ± 0.5^a^	6.6 ± 0.5^a^	6.7 ± 0.5^a^	7.1 ± 0.5^a^
	0.5 μl L^−1^	7.8 ± 0.7^a^	7.1 ± 0.6^a^	6.0 ± 0.5^a^	6.4 ± 0.6^a^	6.7 ± 0.5^a^
20°C 5 days	0 μl L^−1^	—	—	—	—	—
	0.25 μl L^−1^	—	—	—	—	—
	0.5 μl L^−1^	7.3 ± 0.3	6.7 ± 0.5	6.2 ± 0.6	7.6 ± 0.5	7.3 ± 0.6

Data are the means of nine replicates ± standard deviation. In columns, different superscript letters at the same time indicate significant differences according to Duncan’s test (*p* < 0.05).

Before treatment with 1-MCP, the pulp firmness, SSC, TA, BAR, starch, and h° of ripe kiwifruit were 10.98 N, 14.5 1%, 1.03%, 13.89, 6.41 g kg^−1^, and 105.13 (Figures 4B–F), respectively. As expected, the pulp firmness of the 1-MCP treatment showed a slowly decreasing trend during the shelf life, and the pulp firmness of the 0.5 μl L^−1^ 1-MCP group was higher than that of the other groups from 14 days (4°C) to 7 days (20°C) (Figure 4B). The SSC of the 0 μl L^−1^ 1-MCP group increased rapidly until 5 days (20°C) and then decreased slowly, while the SSC of the 0.25 and 0.5 μl L^−1^ 1-MCP groups increased much slower than that of the 0 μl L^−1^ 1-MCP group (Figure 4C). The highest SSCs of the three groups were 16.57%, 16.70%, and 16.55% before decay (Figure 4C). For TA, there was no difference between the 0.25 and 0.5 μl L^−1^ 1-MCP groups at 14 days (4°C), but the TA of the 0.25 μl L^−1^ 1-MCP group was higher than that of the 0 and 0.5 μl L^−1^ 1-MCP groups at 20°C. The BAR of the 0.5 μl L^−1^ 1-MCP group was lower than that of the 0 and 0.25 μl L^−1^ 1-MCP groups until 5 days (20°C) due to the highest SSC and lowest TA (Figure 4D). Because of the increased SSC (Figure 4C), the BAR of the 0.5 μl L^−1^ 1-MCP group increased rapidly at 3 days (20°C) and reached the highest level (18.86%) at 5 days (20°C). According to the BAR and decay percentage, the quality of kiwifruit treated with 0.5 μl L^−1^ 1-MCP was better than that of the 0 and 0.25 μl L^−1^ 1-MCP groups.

In a previous study, the starch content was positively correlated with pulp firmness. The decreasing trend of starch content was similar to that of pulp firmness, as shown in Figure 4E, so the 0.5 μl L^−1^ 1-MCP group was slower than the groups of 0 and 0.25 μl L^−1^ 1-MCP from 0 to 5 days (20°C). In this study, there was a rapid decrease in starch content in the CK and M1 groups on the 4°C shelves. The starch contents of the 0, 0.25, and 0.5 0.5 μl L^−1^ 1-MCP groups were 0.83, 0.84, and 0.70 g kg^−1^ before decay, respectively. As a result, 0.5 μl L^−1^ 1-MCP treatment delayed the starch degradation of ripe kiwifruit.

According to a study on d'Anjou, 1-MCP treatment delayed the decrease in h° so that the peel color was greener than that of the control group (Guo et al., 2020). In this study, 1-MCP effectively delayed the h° decrease in ripening kiwifruit, which was higher than 0 μl L^−1^ 1-MCP group from 14 days (4°C) to 7 days (20°C) (Figure 4F). This suggested that 0.5 L L^−1^ 1-MCP could keep the pulp color of “Guichang” kiwifruit.

**FIGURE 5:**
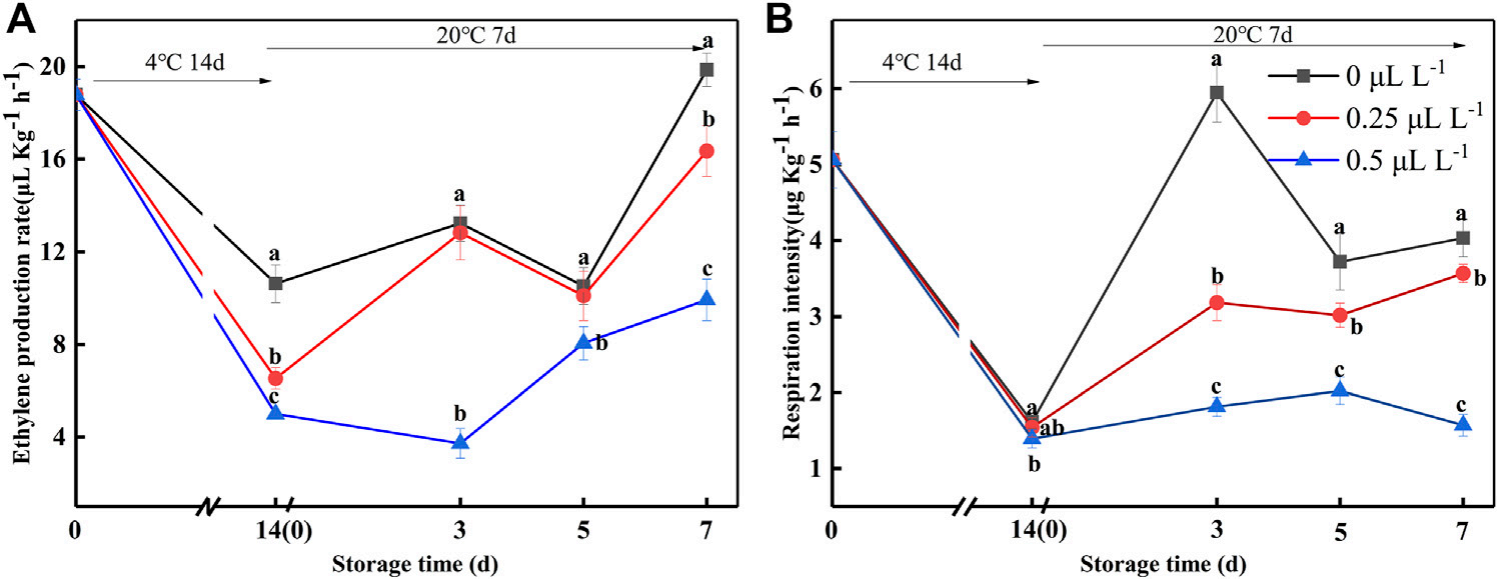
Ethylene production rate **(A)** and respiration intensity **(B)** variations during shelf life in the 1-MCP preservation experiment. Data are the means of three replicates ± standard deviation (*n* = 18). According to Duncan's test, values with different letters are significantly different (*p* < 0.05).

To verify the effectiveness of 1-MCP on ripening fruits, EP and RI were measured (Figure 5). The EP and RI were 6.57 ng kg^−1^ s^−1^ and 1.41 ng kg^−1^ s^−1^ at 0 day, respectively. The EP and RI of the 0, 0.25, and 0.5 μl L^−1^ 1-MCP groups showed a similar trend: they decreased at 14 days (4°C) but rapidly increased at 3 days (20°C). Throughout the 20°C shelf life, the EP and RI of the 0.5 μl L^−1^ 1-MCP group were lower than those of the 0 and 0.25 μl L^−1^ 1-MCP groups. Hence, the 1-MCP treatment effectively inhibited the increase in EP and RI at both 4 and 20°C.

**FIGURE 6:**
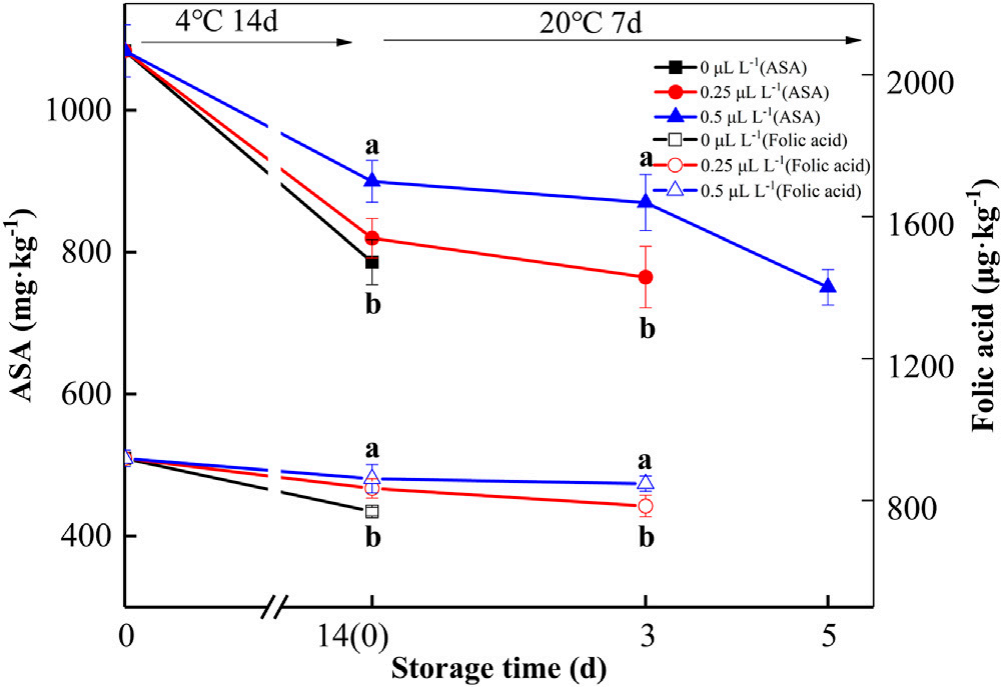
ASA and folic acid variations during shelf life in the 1-MCP preservation experiment. Data are the means of three replicates ± standard deviation (*n* = 18). According to Duncan’s test, values with different letters are significantly different (*p* < 0.05).

ASA and folic acid are important nutrients, and 1-MCP can inhibit the metabolism of ASA and folic acid in kiwifruit (Xu et al., 2019). At 0 day, the ASA and folic acid contents of the sample were 1,083.8 mg kg^−1^ and 918.4 μg kg^−1^, respectively, as shown in Figure 6. The ASA and folic acid contents gradually decreased over time. The ASA and folic acid contents of 0, 0.25, and 0.5 μl L^−1^ 1-MCP groups were 545.1, 637.6, 750.7 mg kg^−1^ and 606.1, 654.5, and 764.8 μg kg^−1^ at 7 days (20°C), respectively. Hence, 0.5 μl L^−1^ 1-MCP treatment effectively delayed the decline in ASA and folic acid in kiwifruit.

### 3.4 The Flavor Compounds of Ready-to-Eat Kiwifruit

Flavor is a fundamental field that includes volatiles and taste (sugars and acids), which are detected in the mouth and nose and affect the eating quality of kiwifruit (Civille and Oftedal, 2012; Zeng et al., 2020). Previous research finds that ethylene ripening improved kiwifruit flavor (sweetness and aroma) (Günther et al., 2015; Lim et al., 2017). However, the effect of 1-MCP on the flavor of “edible window” kiwifruit was not clear. There was no significant difference in BAR (Figure 4D) before decay, in this study, but the “fruit” aroma from the sensory evaluation (Table 1) was insufficient after 0.5 μl L^−1^ 1-MCP treatment. Thus, the current study compared the changes in volatile components between ready-to-eat kiwifruit and kiwifruit subjected to ethylene ripening.

**FIGURE 7:**
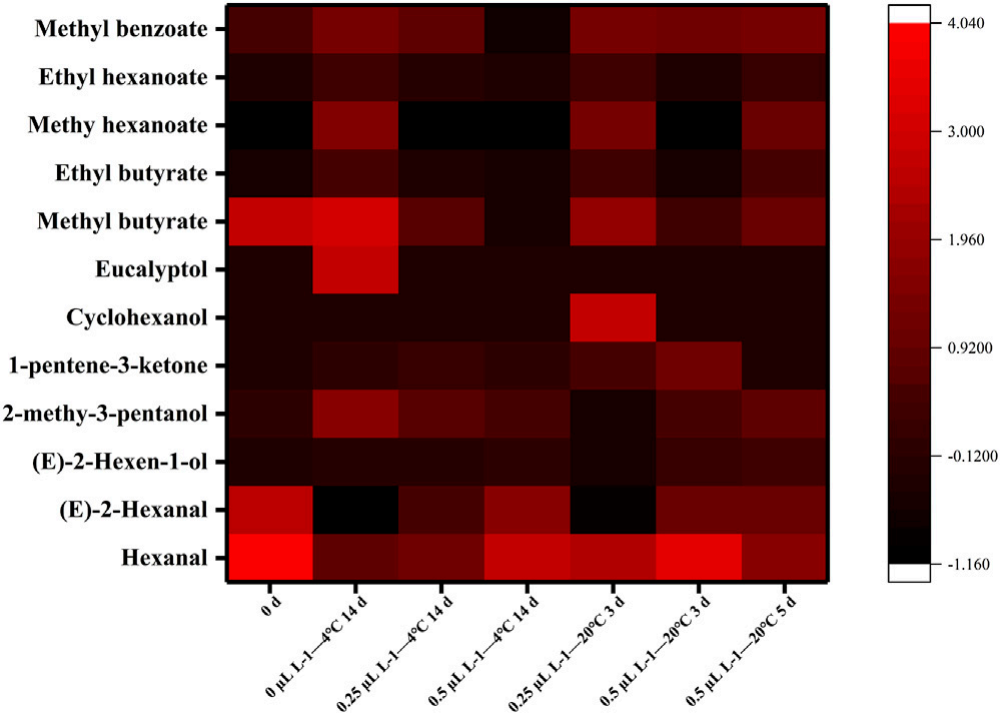
Heatmap of volatile components.

**FIGURE 8:**
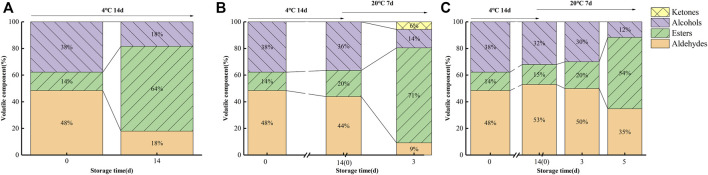
Proportions of aldehyde, ester, alcohol, and ketone variations during storage at 4 and 20°C (**(A)** CK; **(B)** M1; **(C)** M2).

Figures 7 and 8 illustrate the differences in kiwifruit scent in each group. Chen et al. concluded that the volatile components of kiwifruit at lower maturity were mainly aldehydes. Then, esters were gradually formed when the fruit ripened (Chen et al., 2020). Volatile chemicals are represented by colors in the heatmap (Figure 7); the redder the color, the higher the amount. The contents of hexanal and (*E*)-2-hexanal were the highest in sample 0 days. In contrast, they were lowest in sample 0 μl L^−1^ −4°C at 14 days and lower than samples 0.25 μl L^−1^ −20°C at 3 days and 0.5 μl L^−1^ −20°C at 5 days. The content of eucalyptol was the highest in sample 0 μl L^−1^ −4°C at 14 days. The contents of cyclohexanol and 1-pentene-3-ketone were the highest in the sample 0.25 μl L^−1^ −20°C at 3 days. At 14 days, sample 0 μl L^−1^ at −4°C included greater concentrations of methyl butyrate, ethyl butyrate, methyl hexanoate, methyl benzoate, and ethyl hexanoate. Consequently, the 1-MCP treatment effectively inhibited the production of these ester substances. Combined with the results from Figures 7 and 8, it is clear that the aldehyde concentration declined over time, but the ester content rose. In 14 days (4°C), the proportions of aldehydes, esters, and alcohols in the 0 μl L^−1^ 1-MCP group were 18, 64, and 18%, respectively. Meanwhile, there were 44%, 20%, and 36% in the 0.25 μl L^−1^ 1-MCP group and 56%, 8%, and 36% in the 0.5 μl L^−1^ 1-MCP group. This finding revealed that 1-MCP hindered the breakdown of aldehydes and alcohols or the creation of esters, which corroborated Zhang et al.’s (2009) report. On the other hand, the proportion of esters in the 0.5 μl L^−1^ 1-MCP group (20%) was lower than that in the 0.25 μl L^−1^ 1-MCP group (71%) at 20°C for 3 days. However, it increased to 54% at 20°C for 5 days. Hence, the inhibitory effect of 0.5 μl L^−1^ 1-MCP on the “fruit” aroma was more obvious. This outcome was consistent with the sensory evaluation results. The aroma value of the 0.5 μl L^−1^ 1-MCP group was lower than that of the 0 and 0.25 μl L^−1^ 1-MCP groups before 20°C for 5 days, but there was no difference before decay (Table 1). The exact composition of the volatile component is presented in Supplementary Table S2.

### 3.5 Multivariate Analysis for the Eating Quality of Ready-to-Eat Kiwifruit

To obtain a more comprehensive assessment of ready-to-eat kiwifruit eating quality, the indicators of BAR, pulp firmness, h°, “overall” of sensory evaluation, ASA, folic acid, hexenal, *(E)*-2-hexenal, methyl butyrate, and ethyl butyrate were selected and analyzed using PCA and HCA. Thus, the edible quality at different stages was clearly defined.

**FIGURE 9:**
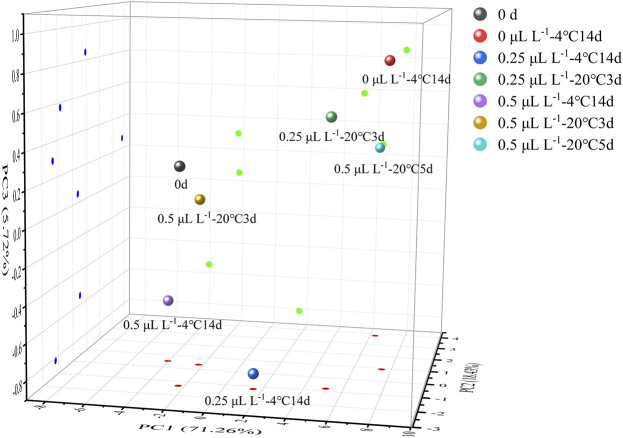
3D projection plots of PCA are based on the three principal components of the nine samples.

Figure 9 illustrates the PCA results. PC1, PC2, and PC3 contributed 71.26%, 18.43%, and 5.72%, respectively, to the variance, with the three principal components accounting for 95.41 percent. Color, aroma, taste, and nutrition are important sensory indicators for measuring food quality for consumers. From the results of the sensory evaluation, sample 0 μl L^−1^ −4°C at 14 days was used as a standard because it had the highest value of “overall” in Table 1. Clearly, sample 0.25 μl L^−1^ −20°C at 5 days was closest to sample 0 μl L^−1^ −4°C at 14 days. This indicated that the eating quality was comparable to sample 0 μl L^−1^ −4°C at 14 days. By contrast, sample 0 day was closer to sample 0.5 μl L^−1^ −4°C at 14 days and 0.5 μl L^−1^ −20°C at 3 days. This indicated that μl L^−1^ 1-MCP delayed the change in eating quality. However, prior to decay, the eating quality was similar between ethylene-ripening kiwifruit and 0.5 μl L^−1^ 1-MCP-treated kiwifruit. HCA was used to confirm the PCA results. All samples were divided into two clusters: samples 0 day, 0.25 μl L^−1^ −4°C at 14 days, 0.25 μl L^−1^ −20°C at 3 days, 20°C at 5 days, 0.5 μl L^−1^ −4°C at 14 days, 0.5 μl L^−1^ −20°C at 3 days, and 0.5 μl L^−1^ −20°C at 5 days in Cluster I and samples 0 μl L^−1^ −4°C at 14 days and 0.5 μl L^−1^ −20°C at 5 days in cluster II. Significantly, the Euclidean distance between 0 μl L^−1^ −4°C at 14 days and 0.5 μl L^−1^ −20°C at 5 days was minimal. The result was provided for PCA. Although the “overall” value of all groups showed no significant difference before decay (Table 1), the eating quality of 0.5 μl L^−1^ −20°C at 5 days was most similar to that of 0 μl L^−1^ −4°C at 14 days.

## 4 Discussion

As a climacteric fruit, kiwifruit ripens slowly under natural conditions and begins to deteriorate rapidly when climacteric production occurs, greatly affecting fresh kiwifruit sales and exacerbating post-harvest fruit loss. This trial establishes that the sequential applications of ethylene and 1-MCP to inedible kiwifruit could accelerate its after-ripening and maintain the “edible window.” However, it is necessary to first determine the threshold of 1-MCP on kiwifruit. It is attempting to avert an adverse situation that occurs when excessive fruit maturity results in 1-MCP ineffectively. 1-MCP is an ethylene receptor blocker that protects the fruit from the interference of ethylene and delays the deterioration of fruit quality (Gong et al., 2020). In this study, 1-MCP was unable to inhibit the ethylene production rate and respiration intensity of kiwifruit when SSC exceeded 15%. Meanwhile, the firmness of the fruit rapidly decreased, and the decay percentage was over 47.91%. To further improve the ripening effectiveness, this study applied higher concentrations of ethylene (100–1,000 μl L^−1^) to kiwifruit. As expected, the ethylene treatment had a noticeable effect. The ethylene production rate and respiration intensity of all ethylene-treated groups rapidly increased, with rapid changes in pulp firmness, SSC, BAR, h°, and starch content. However, the 1,000 and 2,000 μl L^−1^ ethylene treatments performed poorly in terms of ripening efficiency. When the EP and RI (Figure 3) reached the maximum, the fruit of the ET groups began to decay (Figure 2A), which indicated that the fruit was in senescence. Taken together, there were no significant differences between the ET groups in terms of pulp firmness, SSC, TA, or h°. In a previous study, in which it took at least 2 days to reach edible hardness (Park et al., 2006; Lim et al., 2017), the fruit of the 250 μl L^−1^ ethylene-treatment group was close to edible firmness at 1 day. The BAR of the E2 group was the highest before decay in the ET groups (Figures 2B–D). Additionally, the starch content of E1, E3, E4, and E5 was lower than that of E2 after 3 days (Figure 2E), indicating that the quality of E1, E3, E4, and E5 was lower than that of E2, despite the fact that they were within the “edible window.” As a result, it was recommended that “Guichang” kiwifruit should be treated with 250 μl L^−1^ ethylene. However, the edible kiwifruit must be consumed within 5 days (Figure 3A), which presents a challenge for both sellers and consumers. After ripening, it is necessary to preserve the “Guichang” kiwifruit.

After 1 day of 250 μl L^−1^ ethylene ripening, the maturity of the fruit did not exceed the 1-MCP threshold (SSC: 15%). In this case, 1-MCP could be used immediately to treat the fruit. As illustrated in Figure 4, 0.5 μl L^−1^ 1-MCP treatment inhibited the decay percentage. In addition, the changes in pulp firmness, SSC, BAR, starch, ASA, and folic acid content were suppressed. 1-MCP treatment had no effect on the overall pattern of pulp firmness reduction; however, it slowed the rate of pulp firmness reduction. In the present study, the pulp firmness of 0.5 μl L^−1^ 1-MCP-treated fruits was close to CK at 20°C for 7 days. Furthermore, the changes in SSC were the same.

While 1-MCP treatment is an effective way to delay the deterioration of fruit quality and extend its shelf life, its misuse will result in a loss of flavor (Huan et al., 2020). Flavor consists of BAR and volatile compounds. In this study, as the TA content of fruits in the 0.5 μl L^−1^ 1-MCP treatment group was effectively maintained, BAR was at the lowest level. However, a comprehensive decay percentage analysis revealed no difference in the maximum BAR of fruits before decay between any of the two groups. On the other hand, there was no difference in the highest score of “taste” among all groups. Aldehydes and esters are important volatile components in kiwifruit that contribute to a special aroma. In this study, the predominant aldehydes were *(E)*-2-hexenal and hexenal, which were characteristic volatile components of kiwifruit and contributed to its “grassy” aroma (Du et al., 2019; Lan et al., 2021). The “sweetness” and “fruit” aromas in kiwifruit were attributed to esters such as methyl butyrate, ethyl butyrate, and methyl hexanoate. In addition, the typical kiwifruit aroma was produced by ethyl butyrate (Zhao et al., 2021). During late ripening, the content of ester was increased. As shown in Figure 7, the content of ester of 1-MCP-treated fruit was significantly lower than that of CK. 1-MCP did not completely inhibit the production of esters. According to Figures 7 and 8, the changing trends of fruit ester content in all groups were the same, and the rate of the 1-MCP treatment group was lower than that of the CK group solely. The percentages of aldehydes and esters in the fruits of all groups were very similar before decay.

The edible quality of 0.5 μl L^−1^ 1-MCP-treated fruit was comparable to that of the CK group. Although the samples from the 0.5 μl L^−1^ 1-MCP group began to decay at 20°C for 7 days, the decay percentage was the lowest (5.56%). This meant that more fruits with higher eating quality could maintain a longer “edible window” in the 0.5 μl L^−1^ 1-MCP group. The flavor and taste of 0.5 μl L^−1^ 1-MCP-treated fruits were worse than those of CK. However, due to the longer “edible window” of ready-to-eat kiwifruit, its flavor and taste would gradually improve. In summary, the optimal 1-MCP treatment concentration of ready-to-eat kiwifruit was 0.5 μl L^−1^.

Recently, the kiwifruit industry has been severely impacted by the limiting factors of slow ripening and subsequent rapid deterioration. In previous studies, kiwifruit was always treated with ethylene or 1-MCP alone. Innovatively, ethylene ripening followed by 1-MCP preservation was employed in the present study. Finally, ready-to-eat kiwifruit was prepared by the regulation of ethylene and 1-MCP. A simple and low-cost strategy was attempted and confirmed to extend the “edible window.” Ready-to-eat “Guichang” kiwifruit was prepared using 250 μl L^−1^ ethylene (20°C, 36 h), followed by 0.5 μl L^−1^ 1-MCP for 24 h (20°C), resulting in a longer shelf life (4°C for 14 days, 20°C for 5 days) than the CK group (4°C for 14 days). We hope that ready-to-eat kiwifruit will be a viable alternative to traditional marketing and production patterns of the climacteric fruit industry.

## Data Availability

The original contributions presented in the study are included in the article/Supplementary Material; further inquiries can be directed to the corresponding author.

## Supplementary Material

The Supplementary Material for this article can be found online at: https://www.frontiersin.org/articles/10.3389/fchem.2022.934032/full#supplementary-material .
