# Supplementary material for: Preparation, Shelf, and Eating Quality of Ready-to-Eat “Guichang” Kiwifruit: Regulation by Ethylene and 1-MCP
Source: Front Chem. 2022 Jul 13;10:934032. doi: 10.3389/fchem.2022.934032 (PMC9326346; doi:10.3389/fchem.2022.934032)
Supplement: Supplementary file 1 [file DataSheet1.docx]

**Supplementary** **Material**

**FIGURE S1.** O_2_ concentrations (**A**) and CO_2_ concentrations (**B**) variations in boxes. (CK: 0 μL L^-1^, E1: 100 μL L^-1^, E2: 250 μL L^-1^, E3: 500 μL L^-1^, E4: 1000 μL L^-1^, E5: 2000 μL L^-1^). Data are the means of three replicates ± standard deviation (n=18). According to Duncan's test, values with different letters are significantly different (*P*< 0.05).

**Table S2.** Volatile component (μg/Kg) of kiwifruit with 1-MCP treatment.

**FIGURE S3.** Clustering dendrogram of the kiwifruit samples using Euclidean distance.





**FIGURE S1.** O_2_ concentrations (**A**) and CO_2_ concentrations (**B**) variations in boxes. (CK: 0 μL L^-1^, E1: 100 μL L^-1^, E2: 250 μL L^-1^, E3: 500 μL L^-1^, E4: 1000 μL L^-1^, E5: 2000 μL L^-1^). Data are the means of three replicates ± standard deviation (n=18). According to Duncan's test, values with different letters are significantly different (*P*< 0.05).

**TableS****2.** Volatile component (μg kg^-1^) of kiwifruit with 1-MCP treatment

| NO. | Constituents | group | Time | | | | |
| --- | --- | --- | --- | --- | --- | --- | --- |
|  |  |  | 0 d | 4 ℃ 14 d | 20 ℃ 3 d | 20 ℃ 5 d |  |
|  | Aldehydes |  |  |  |  |  |  |
| 1 | Hexanal | 0 μL L^-1^ | 48.34±4.92^b^ | 20.65±2.42^b^ | - | - |  |
|  |  | 0.25 μL L^-1^ |  | 21.38±2.27^b^ | ND | - |  |
|  |  | 0.5 μL L^-1^ |  | 141.54±15.34^a^ | 106.73±8.52 | 99.8±8.68 |  |
| 2 | (E)-2-Hexenal | 0 μL L^-1^ | 133.46±6.23^b^ | 156.19±15.64^b^ | - | - |  |
|  |  | 0.25 μL L^-1^ |  | 176.46±19.5^b^ | 97.81±7.63^b^ | - |  |
|  |  | 0.5 μL L^-1^ |  | 214.45±22.94^a^ | 236.13±16.23^a^ | 298.63±22.79 |  |
|  | Esters |  |  |  |  |  |  |
| 3 | Methyl butyrate | 0 μL L^-1^ | 50.07±1.12^a^ | 83.68±4.66^a^ | - | - |  |
|  |  | 0.25 μL L^-1^ |  | 88.79±13.56^a^ | 103.50±10.48^a^ | - |  |
|  |  | 0.5 μL L^-1^ |  | 52.14±7.16^b^ | 26.81±13.83^b^ | 68.78±7.33 |  |
| 4 | Ethyl butyrate | 0 μL L^-1^ | ND | 542.16±38.68 | - | - |  |
|  |  | 0.25 μL L^-1^ |  | ND | 651.70±40.70 | - |  |
|  |  | 0.5 μL L^-1^ |  | ND | ND | 544.32±43.57 |  |
| 5 | Methyl hexanoate | 0 μL L^-1^ | ND | 75.64±16.44 | - | - |  |
|  |  | 0.25 μL L^-1^ |  | ND | 43.11±3.26 | - |  |
|  |  | 0.5 μL L^-1^ |  | ND | ND | 41.69±4.06 |  |
| 6 | Ethyl hexanoate | 0 μL L^-1^ | ND | 103.65±11.35^a^ | - | - |  |
|  |  | 0.25 μL L^-1^ |  | 49.72±6.59^b^ | 98.44±7.88^a^ | - |  |
|  |  | 0.5 μL L^-1^ |  | ND | 72.33±9.11^b^ | 84.16±9.75 |  |
| 7 | Methyl benzoate | 0 μL L^-1^ | 2.03±0.21 | 0.80±0.08 | - | - |  |
|  |  | 0.25 μL L^-1^ |  | ND | 3.35±0.34^a^ | - |  |
|  |  | 0.5 μL L^-1^ |  | ND | 4.42±0.97^a^ | 1.68±0.61 |  |
|  | Alcohols |  |  |  |  |  |  |
| 8 | *(E)*-2-Hexen-1-ol | 0 μL L^-1^ | 48.52±4.55 | 72.99±5.47^b^ | - | - |  |
|  |  | 0.25 μL L^-1^ |  | 74.47±9.37^b^ | 28.02±4.55^b^ | - |  |
|  |  | 0.5 μL L^-1^ |  | 141.49±18.50^a^ | 67.24±6.28^a^ | 76.80±7.03 |  |
| 9 | Cyclohexanol | 0 μL L^-1^ | ND | 24.00±4.02 | - | - |  |
|  |  | 0.25 μL L^-1^ |  | ND | ND | - |  |
|  |  | 0.5 μL L^-1^ |  | ND | ND | ND |  |
| 10 | 2-methyl-3-pentanol | 0 μL L^-1^ | 84.44±9.11^a^ | 82.09±11.09^a^ | - | - |  |
|  |  | 0.25 μL L^-1^ |  | 89.93±7.32^a^ | 115.17±11.28^b^ | - |  |
|  |  | 0.5 μL L^-1^ |  | 83.79±2.71^a^ | 159.57±18.35^a^ | 57.16±10.07 |  |
| 11 | Eucalyptol | 0 μL L^-1^ | 8.92±1.42^a^ | 11.79±0.24 | - | - |  |
|  |  | 0.25 μL L^-1^ |  | ND | 4.32±0.73^a^ | - |  |
|  |  | 0.5 μL L^-1^ |  | ND | 2.52±0.41^b^ | ND |  |
|  | Ketones |  |  |  |  |  |  |
| 12 | 1-pentene-3-ketone | 0 μL L^-1^ | ND | ND | - | - |  |
|  |  | 0.25 μL L^-1^ |  | ND | 60.42±3.81 | - |  |
|  |  | 0.5 μL L^-1^ |  | ND | ND | ND |  |

**
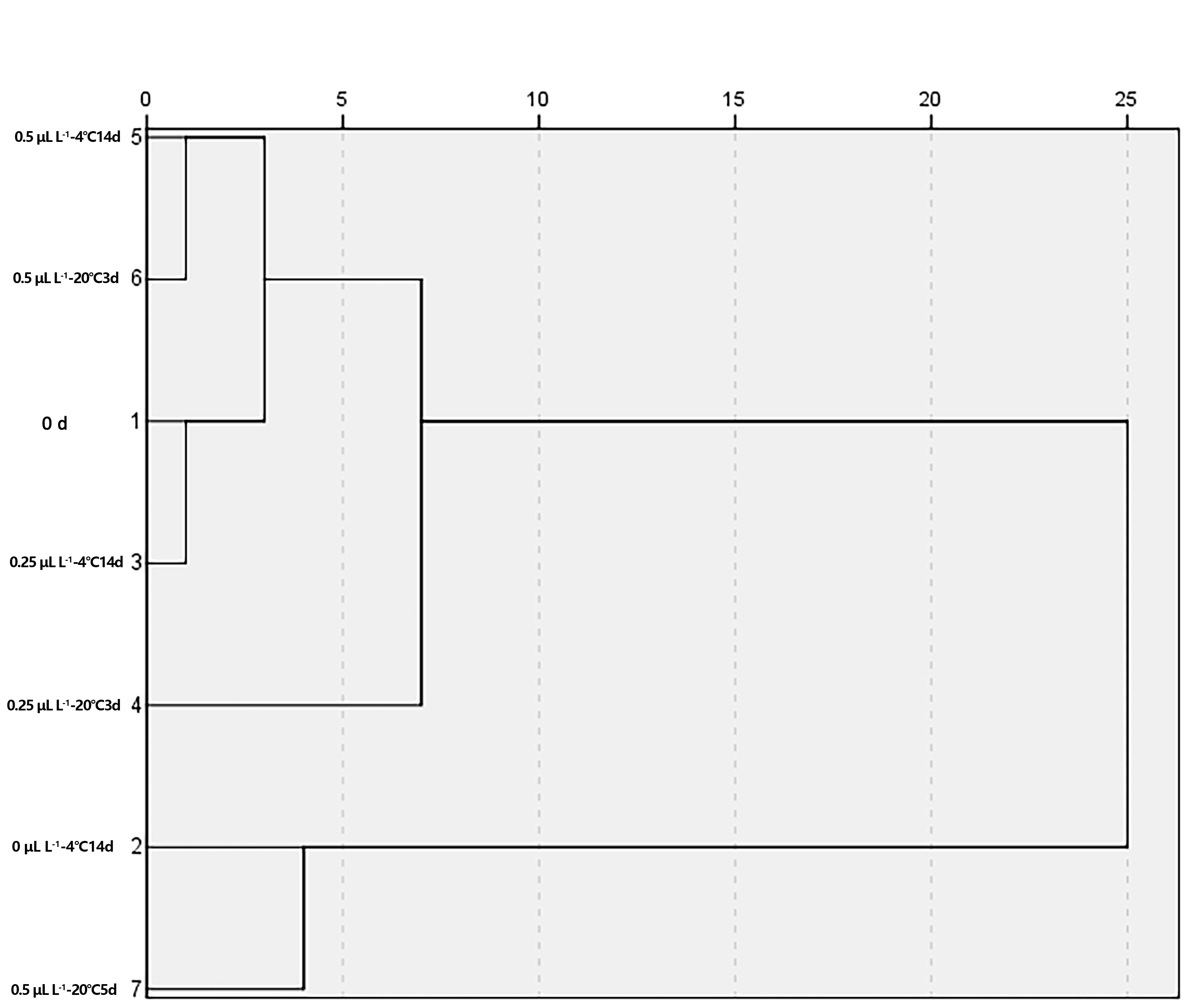
**

**FIGURE S3.** Clustering dendrogram of the kiwifruit samples using Euclidean distance.
